# Scutellarin attenuates hypoxia/reoxygenation injury in hepatocytes by inhibiting apoptosis and oxidative stress through regulating Keap1/Nrf2/ARE signaling

**DOI:** 10.1042/BSR20192501

**Published:** 2019-11-13

**Authors:** Haiyuan Wu, Lan Jia

**Affiliations:** Department of Pathology, Shanxian Central Hospital, Shanxian 274300, Shandong Province, China

**Keywords:** hepatic ischemia-reperfusion (I/R) injury, hepatocytes, Keap1/Nrf2/ARE signaling pathway, oxidative stress, reactive oxygen species (ROS), scutellarin

## Abstract

Scutellarin is a natural flavonoid that has been found to exhibit anti-ischemic effect. However, the effect of scutellarin on hepatic hypoxia/reoxygenation (ischemia–reperfusion (I/R)) injury remains unknown. The aim of the present study was to explore the protective effect of scutellarin on I/R-induced injury in hepatocytes. Our results showed that **s**cutellarin improved cell viability in hepatocytes exposed to hypoxia/reoxygenation (H/R). Scutellarin treatment resulted in decreased levels of reactive oxygen species (ROS) and malondialdehyde (MDA), and increased superoxide dismutase (SOD) activity in H/R-induced hepatocytes. In addition, scutellarin reduced cell apoptosis in H/R-stimulated hepatocytes, as proved by the decreased apoptotic rate. Moreover, scutellarin significantly up-regulated bcl-2 expression and down-regulated bax expression in hepatocytes exposed to H/R. Furthermore, scutellarin treatment caused significant decrease in Keap1 expression and increase in nuclear Nrf2 expression. Besides, scutellarin induced the mRNA expressions of heme oxygenase-1 (HO-1) and NAD(P)H:quinone oxidoreductase 1 (NQO1). Inhibition of Nrf2 significantly reversed the protective effects of scutellarin on H/R-stimulated hepatocytes. In conclusion, these findings demonstrated that scutellarin protected hepatocytes from H/R-induced oxidative injury through regulating the Keap1/Nrf2/ARE signaling pathway, indicating a potential relevance of scutellarin in attenuating hepatic I/R injury.

## Introduction

Hepatic ischemia–reperfusion (I/R) injury is a commonly identified clinical complication that frequently occurs after liver transplantation, liver resection, stroke, and trauma, which may result in organ dysfunction and failure [1,2]. Therefore, it is generally believed that I/R injury is negatively associated with the survival rate and prognosis of the patients. Hepatic I/R injury remains a major unsolved public health problem since there is still a lack of effective therapeutics for preventing and treating hepatic I/R injury [3].

During the process of hepatic I/R injury, cellular hypoxia and reoxygenation are two essential elements [4]. Ischemic insult not only incurs in direct cellular damage, exacerbation of hypoxic injury after reoxygenation is a more important reason of cellular injury [4]. Convincing evidence has confirmed a variety of molecular mechanisms to explain this phenomenon. Most importantly, excess production of reactive oxygen species (ROS) is considered as a critical factor in the genesis of reperfusion injury. The imbalance between the rate of generation of ROS and the ability to detoxify ROS may lead to oxidative stress and also evoke acute inflammatory response that will further aggravate hepatocellular injury [5]. As a consequence, considerable efforts have been devoted to developing effective agents for blocking the sources of excess ROS production [6].

Scutellarin is a flavonoid derived from *Erigeron breviscapus* (Vant.) Hand.-Mazz., which is a traditional Chinese herbal medicine [7]. The systematic studies of scutellarin in modern medicine proved that scutellarin has multiple pharmacological effects, such as anti-inflammatory and antioxidative activity, anti-apoptosis, anti-diabetic, anti-ischemic, anti-cancer effect, anti-neurodegeneration, and anti-glaucoma effect [7–10]. Therefore, the multi-effective nature of scutellarin suggests that it possesses potential clinical applications for the treatment of diverse diseases including tissues I/R injury [11–13]. However, the effect of scutellarin on hepatic I/R injury remains unclear. Thus, in the present study, we examined the effect of scutellarin on hepatic hypoxia/reoxygenation (H/R) injury *in vitro*.

## Materials and methods

### Cell culture

Human hepatocyte line HL-7702 (Cell Bank of Shanghai Institutes for Biological Sciences, Chinese Academy of Sciences, Shanghai, China) was maintained in RPMI-1640 medium (Hyclone, Logan, UT, U.S.A.) supplemented with 10% (v/v) fetal bovine serum (FBS) and antibiotics (100 U/ml penicillin and 100 μg/ml streptomycin) in an atmosphere containing 5% CO_2_ at 37°C.

### H/R model

The RPMI-1640 medium was replaced by glucose/serum-free medium before hypoxia. Then the cells were cultured under hypoxic condition (containing 1% O_2_) for 4 h. Subsequently, 4 h of reoxygenation was achieved by incubating under a normal condition (containing 95% air and 5% CO_2_) and the glucose/serum in the medium was restored.

### Cell viability assay

HL-7702 cells were collected and seeded into 96-well culture plates, followed by an incubation at 37°C in 5% CO_2_ for 24 h. After different indicated treatments and stimulation, MTT (100 μl; 5 mg/ml; Sigma–Aldrich, St. Louis, MO, U.S.A.) was added to each well and incubated for another 4 h. Then the supernatants were discarded, and the formazan crystal was dissolved through incubation with 150 μl DMSO in the dark for 10 min. Optical density (OD) values were read at the wavelength of 490 nm.

### Measurement of ROS production

Since 2′,7′-dichlorfluorescein diacetate (DCFH-DA) can be oxidized to the highly fluorescent 2,7-dichlorofluorescein (DCF) in the presence of ROS, therefore, ROS formation was determined using the fluorescent probe DCFH-DA. The HL-7702 cells were incubated with 10 μM DCFH-DA for 30 min at 37°C in the dark. The cells were then harvested for the detection of fluorescence intensity using a fluorospectrophotometer (Hitachi, Tokyo, Japan) with excitation/emission maxima of 485/525 nm.

### Measurement of mitochondrial ROS by MitoSOXRed

For detection of mitochondrial superoxide generation, MitoSOXRed assay (Invitrogen/Molecular Probes, U.S.A.) was performed. Briefly, after treatment, HL-7702 cells were incubated with 5 μM of MitoSOX Red for 30 min at 37°C. MitoSOX Red fluorescent intensity was determined at 510 nm excitation and 580 nm emission. After incubation, these cells were washed twice with PBS, trypsinized, resuspended, and immediately submitted to flow cytometry analysis.

### Detection of malondialdehyde level and superoxide dismutase activity

At the end of the treatments, HL-7702 cells were collected and lysed with cold PBS, followed by centrifugation at 15000×***g*** for 10 min at 4°C. The cell lysates were centrifuged at 4°C 12000×***g*** for 10 min. The resulting cell lysates were utilized to assess the malondialdehyde (MDA) content and superoxide dismutase (SOD) activity, using commercially test kits (Nanjing Jiancheng Bioengineering Institute, Nanjing, China) according to the manufacturer’s protocols.

### Western blot

Western blot analysis was performed as described in previous study [14]. Briefly, the obtained whole cell protein extracts and nuclear protein were subjected to 8–12% SDS/PAGE. Subsequently, the resolved protein bands on the gels were transferred on to nitrocellulose membranes, and then blocked with 5% non-fat milk at room temperature for 1 h. Then the immunoblotting analysis was performed using specific antibodies against Keap1, nuclear Nrf2, Nrf2, β-actin, and Lamin B2 (diluted in 1:500; Abcam, Cambridge, MA, U.S.A.) and the following HRP–conjugated secondary antibody (diluted in 1:3000; Abcam). Then the bands were detected using an ECL Western blot substrate (Pierce, Rockford, IL, U.S.A.). The band intensities were quantified using ImageJ gel analysis software (National Institutes of Health, NIH, Bethesda, MD, U.S.A.).

### RNA isolation and quantitative real-time PCR

The total RNA was extracted from tissues and cells with TRIzol reagent (Life Technologies, Scotland, U.K.) according to the manufacturer’s protocol. Then, 3 μg of total RNA was used for the reverse transcription using the Prime Script RT Master Mix (TaKaRa, Shiga, Japan). The quantitative real-time PCR (qRT-PCR) amplification was performed using the SYBR Select Master Mix (Applied Biosystems, Foster, CA, U.S.A.) with the cDNA template on the ABI7300 system (Applied Biosystems). The primer sequences were described as follows: heme oxygenase-1 (HO-1) forward, 5′-GAGGAGTTGCAGGAGCTGCT-3′ and reverse, 5′-GAGTGTAAGGACCCATCGGA-3′; NAD(P)H:quinone oxidoreductase 1 (NQO1) forward, 5′-ACTCTCTGCAAGGGATCCAC-3′ and reverse, 5′- TCTCCAGGCGTTTCTTCCAT-3′; β-actin forward, 5′-CATGTTTGAGACCTTCAACAC-3′ and reverse, 5′-CCAGGAAGGAAGGCTGGAA-3′. Relative gene expression was evaluated using 2^−ΔΔ*C*_t_^ method.

### Cell apoptosis assay

The apoptotic rate was measured by flow cytometry using an Annexin V‐fluorescein isothiocyanate (FITC) apoptosis assay kit (BD Biosciences, Franklin Lakes, NJ, U.S.A.). HL-7702 cells were re-suspended in 500 μl binding buffer containing 5 μl Annexin V-FITC and 5 μl PI, followed by an incubation for 20 min at room temperature in the dark. Apoptotic cells were analyzed by flow cytometry on BD FACSCalibur flow cytometer (BD Biosciences).

### Enzyme linked immunosorbnent assay

After treatment, the levels of oxidative stress-related factors including 3-Nitrotyrosine (3-NT) and 4-Hydroxynonenal (4-HNE) were measured using commercial enzyme linked immunosorbnent assay (ELISA) 3-NT and 4-HNE kits, according to the manufacturer’s instructions.

### Statistical analysis

All experiments were replicated for at least three times. Data are presented as the mean ± standard deviation (SD). Statistical analyses of differences among groups were carried out using GraphPad Prism 5.0 software package (GraphPad Software, Inc., La Jolla, CA, U.S.A.) with the one-way analysis of variance (ANOVA) followed by Tukey’s multiple comparison test. *P*<0.05 was considered to indicate a statistically significant difference.

## Results

### Scutellarin improved the viability of hepatocytes in response to H/R

**Figure 1:**
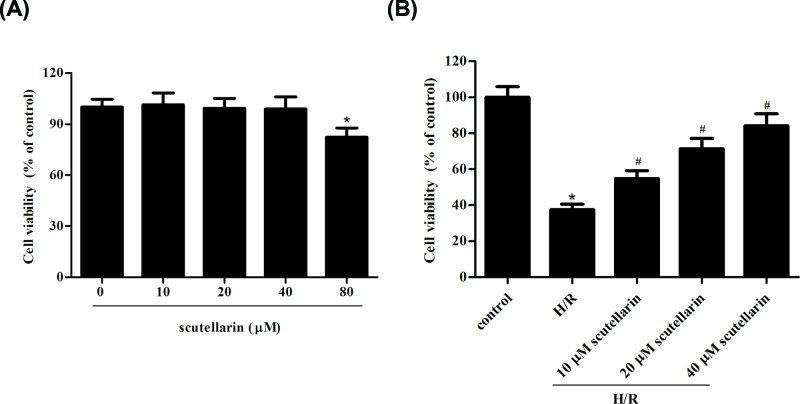
Scutellarin exhibited protective effect on hepatocytes in response to H/R (**A**) Hepatocytes were treated with scutellarin (0, 10, 20, 40 and 80 μM) for 24 h, and cell viability was evaluated using MTT assay. (**B**) Hepatocytes were pre-incubated with scutellarin (10, 20 or 40 μM) for 2 h, followed by H/R stimulation. Then the MTT assay was performed to measure the cell viability of hepatocytes. The data shown are representative of three independent experiments, *n*=5. *P<0.05.

First, we examined the effect of scutellarin on cell viability. The results indicated that scutellarin at the concentration of 80 μM caused a significant inhibition of the viability of hepatocytes. Because administration of scutellarin (10, 20, or 40 μM) had no significant effect on cell viability, these concentrations were selected in the following experiments (Figure 1A). Then, to investigate whether scutellarin could affect the H/R-induced injury in hepatocytes, hepatocytes were pre-incubated with scutellarin (10, 20, or 40 μM) for 2 h, followed by H/R stimulation. Results from MTT assay showed that cell viability was significantly decreased in hepatocytes exposed to H/R stimulation. However, scutellarin pretreatment attenuated the decreased cell viability of H/R-induced hepatocytes (Figure 1B).

### Scutellarin inhibited oxidative stress in H/R-stimulated hepatocytes

**Figure 2:**
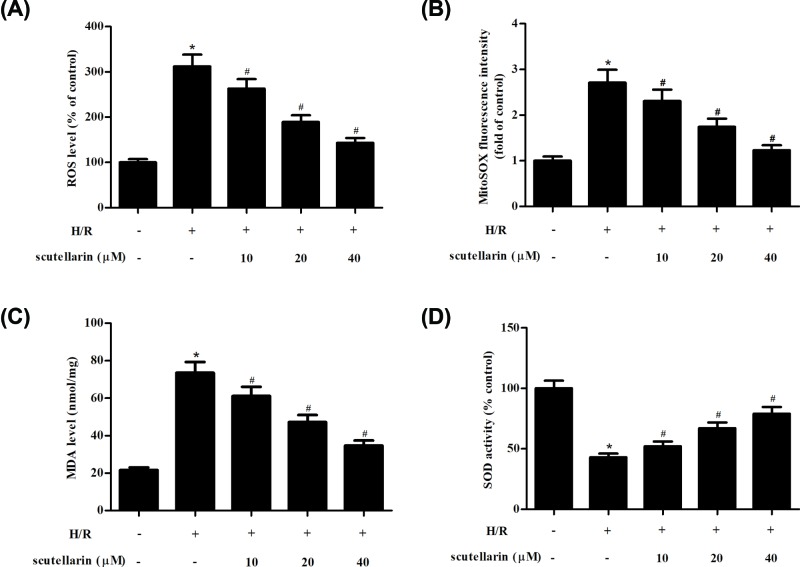
Scutellarin attenuated H/R-stimulated oxidative stress in hepatocytes Hepatocytes were incubated with or without scutellarin (10, 20, or 40 μM) for 2 h, and then subjected to H/R stimulation. The levels of ROS (**A**) and MDA (**C**), as well the SOD activity (**D**) were measured to assess oxidative stress. (**B**) The mitochondria ROS production was detected by MitoSOX Red assay. The data shown are representative of three independent experiments, *n*=5. *P<0.05 vs control group; ^#^*P*<0.05 vs H/R group.

To examine the effect of scutellarin on H/R-induced oxidative stress in hepatocytes, the levels of ROS and MDA, as well the SOD activity were measured. As shown in Figure 2A,C, the levels of ROS and MDA were significantly elevated in H/R-stimulated hepatocytes. While the induction of ROS and MDA production were mitigated by scutellarin. To further explore the source of ROS, MitoSOX Red was used to measure mitochondrial ROS production. As shown in Figure 2B, the ROS in mitochondria was significantly increased in H/R treatment condition. However, the effect was reversed by scutellarin. Moreover, H/R-caused decrease in SOD activity was markedly blocked by scutellarin (Figure 2D and Supplementary Figure S2B). Furthermore, the results of ELISA showed that scutellarin significantly decreased the levels of 3-NT and 4-HNE in H/R-stimulated hepatocytes (Supplementary Figure S1A,B).

### Scutellarin suppressed cell apoptosis induced by H/R in hepatocytes

**Figure 3:**
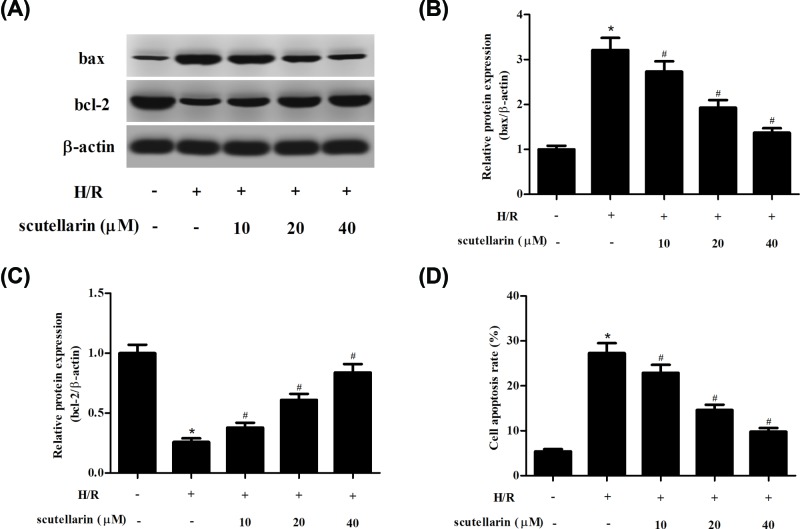
Scutellarin inhibited H/R-induced cell apoptosis in hepatocytes After H/R stimulation in the presence or absence of scutellarin (10, 20 or 40 μM), the effect of scutellarin on cell apoptosis was determined. (**A**) Western blot was applied to detect the expression levels of bax and bcl-2. (**B,C**) Quantification analysis of bax and bcl-2. (**D**) The apoptotic rate was measured by flow cytometry. The data shown are representative of three independent experiments. *n*=4. *P<0.05 vs control group; ^#^*P*<0.05 vs H/R group.

In order to investigate the effect of scutellarin on H/R-induced cell apoptosis in hepatocytes, Western blot was applied to detect the expression levels of bcl-2 and bax. The results showed that scutellarin significantly elevated bcl-2 expression in H/R-stimulated hepatocytes. In contrast, scutellarin down-regulated the H/R-induced bax expression (Figure 3A–C). In addition, cell apoptosis was also measured by flow cytometry. The apoptotic rate in H/R-induced hepatocytes was markedly increased as compared with normal hepatocytes, however, scutellarin reduced the apoptotic rate (Figure 3D).

### Scutellarin regulated the expression of Keap1 and Nrf2 in H/R-stimulated hepatocytes

**Figure 4:**
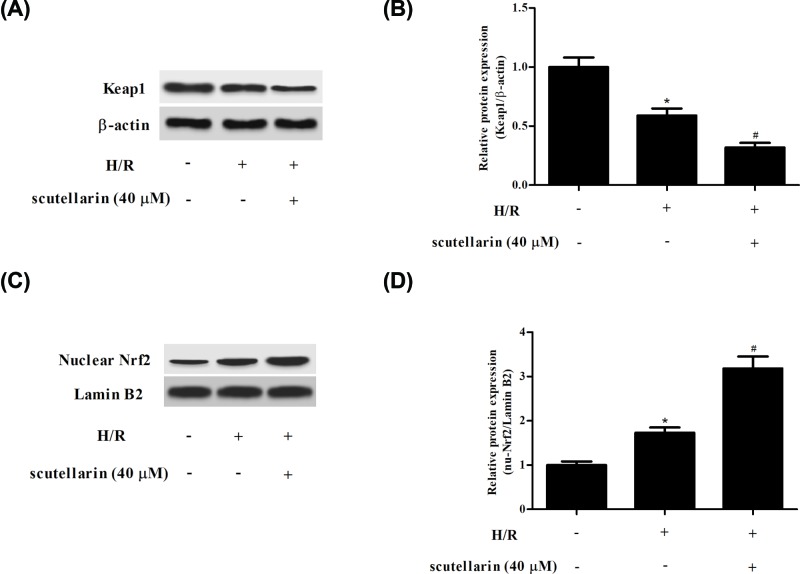
Scutellarin modulated the Keap1/Nrf2 signaling in H/R-stimulated hepatocytes Hepatocytes were incubated with or without scutellarin (10, 20, or 40 μM) for 2 h, and then subjected to H/R stimulation. (**A,C**) The protein expressions of Keap1 and nuclear Nrf2 were measured using Western blot analysis. (**B**) Quantification analysis of Keap1/β-actin. (**D**) Quantification analysis of nuclear Nrf2/Lamin B2. The data shown are representative of three independent experiments; *n*=4. *P<0.05 vs control group; ^#^*P*<0.05 vs H/R group.

Keap1/Nrf2 signaling is associated with the protection of oxidative injury. To uncover the mechanism underlying the scutellarin-mediated protection, the effect of scutellarin on Keap1/Nrf2 signaling was determined. Western blot analysis demonstrated that scutellarin treatment significantly decreased Keap1 expression (Figure 4A,B). Moreover, the nuclear Nrf2 protein expression was markedly increased in scutellarin-treated hepatocytes (Figure 4C,D).

### Scutellarin regulated the activation of ARE pathway in H/R-stimulated hepatocytes

**Figure 5:**
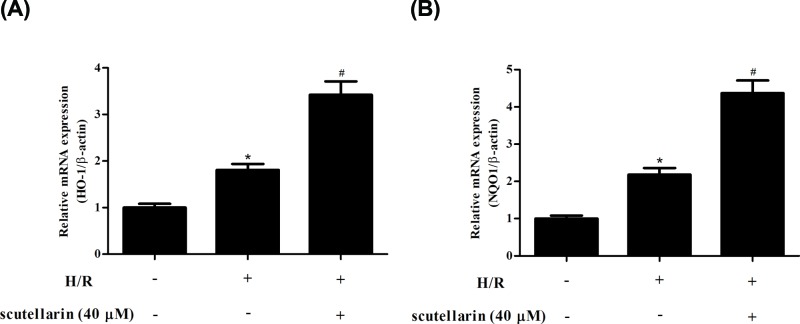
Scutellarin enhanced the H/R-stimulated activation of ARE pathway in hepatocytes Hepatocytes were incubated with or without scutellarin (10, 20, or 40 μM) for 2 h, and then subjected to H/R stimulation. (**A,B**) The mRNA expression levels of downstream genes of ARE pathway, HO-1 and NQO1, were measured using qRT-PCR. The data shown are representative of three independent experiments, *n*=5. *P<0.05 vs control group; ^#^*P*<0.05 vs H/R group.

To explore the activation of ARE pathway, the mRNA expression levels of downstream genes HO-1 and NQO1 were measured using qRT-PCR. As indicated in Figure 5A,B, the increased mRNA expressions of HO-1 and NQO1 caused by H/R stimulation were significantly up-regulated in scutellarin-treated hepatocytes.

### Nrf2 inhibition reversed scutellarin mediated hepato-protective effect in H/R-stimulated hepatocytes

**Figure 6:**
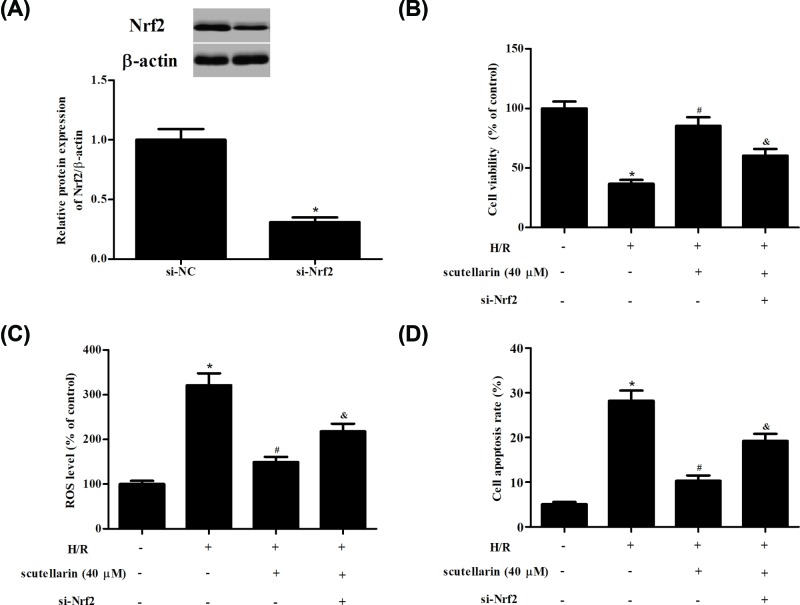
Knockdown of Nrf2 reversed the protective effects of scutellarin in H/R-stimulated hepatocytes Hepatocytes were transfected with siRNA-Nrf2 for 24 h and then subjected to H/R in the presence of 40 μM scutellarin for 24 h. (**A**) Nrf2 protein expression was detected using Western blot. (**B**) Inhibition of Nrf2 reduced cell viability of hepatocytes. (**C**) Inhibition of Nrf2 induced the production of ROS. (**D**) Inhibition of Nrf2 increased apoptotic rate of hepatocytes. The data shown are representative of three independent experiments, *n*=4. *P<0.05 vs control group; ^#^*P*<0.05 vs H/R group; ^&^*P*<0.05 vs H/R+ scutellarin group.

To confirm whether scutellarin exerted its protective effect via regulating the Keap1/Nrf2/ARE signaling pathway, the Nrf2 was knocked down in hepatocytes (Figure 6A). The results showed that inhibition of Nrf2 significantly reversed the protective effects of scutellarin on H/R-stimulated hepatocytes (Figure 6B–D).

## Discussion

In the current study, the results showed that scutellarin exerted protective effect against I/R injury, as evidenced by the elevated cell viability, decreased oxidative stress, and increased cell apoptosis. The underlying mechanism was proved to be associated with its regulatory effect on Keap1/Nrf2/ARE signaling in hepatocytes.

Scutellarin is a natural flavonoid that has been found to exhibit anti-ischemic effect. Scutellarin protects astrocytes from oxygen/glucose-deprived/reperfusion (OGD/R)-induced ischemic injury *in vitro* and attenuates cerebral I/R injury in the rat transient middle cerebral artery occlusion model [15]. The protective effect of scutellarin is attributed to the inhibitory effect on the activity of nicotinamide adenine dinucleotide phosphate oxidase 2 (NOX2), which is responsible for the generation of ROS [15]. Scutellarin protects cardiomyocyte I/R injury by modulating I/R-induced oxidative stress, inflammatory response and apoptosis probably through the JAK2/STAT3 pro-survival signaling pathway [11]. In addition, scutellarin was found to be beneficial in improving bilateral hindlimb I/R-induced lung damage, which is most probably mediated by its antioxidant, anti-inflammatory, and anti-apoptotic effects [16]. However, the effect of scutellarin on hepatic I/R injury remains unknown.

Additionally, scutellarin has been reported to prevent diosbulbin B (DB)-induced liver injury by attenuating NF-κB-mediated hepatic inflammation and liver oxidative stress injury [17]. Scutellarin treatment significantly reduces blood lipid levels and enhances antioxidative capacities in non-alcoholic fatty liver model, indicating that scutellarin possesses strong hypolipidemic, antioxidative, and liver protective activity [18]. These findings suggest that scutellarin executes liver protective effect partially through its antioxidative activity. Therefore, we investigated the role of scutellarin in H/R-induced oxidative damage in hepatocytes. As expected, scutellarin improved cell viability of H/R-induced hepatocytes. Scutellarin exhibited antioxidative activity in response to H/R induction, as proved by the decreased levels of ROS and MDA, and the increased SOD activity. Moreover, we found that scutellarin reduced cell apoptosis of H/R-induced hepatocytes from the decreased apoptotic rate, increased bcl-2 expression and reduced expression of bax. These results implied that scutellarin protected hepatocytes from H/R-induced injury through its antioxidative and anti-apoptotic activities.

Nrf2 is an important transcription factor regulator that plays a crucial role in oxidative stress [19–22]. Hence, the Nrf2 signaling has been considered as therapeutic target of several types of human diseases [23–25]. Emerging evidence has demonstrated that Nrf2 signaling is involved in the pathogenesis of hepatic I/R injury [26]. Kudoh et al. [27] reported that Nrf2 activation protects the liver from I/R injury in mice. Nrf2-deficient livers exhibit enhanced tissue damage after hepatic I/R stimulation. Interestingly, scutellarin has been reported as an enhancer of Nrf2 signaling. Fan et al. [28] demonstrated that scutellarin prevents non-alcoholic fatty liver disease (NAFLD) and hyperlipidemia via attenuation of oxidative stress. The hepatic mRNA expression of HO-1, NQO1, and Nrf2 in scutellarin treatment groups are significantly up-regulated, indicating that the antioxidative effect of scutellarin on NAFLD is dependent on the activation of Nrf2/ARE signaling pathway. Scutellarin exerts hypoglycemic and renal protective effects in db/db mice [29]. Scutellarin inhibits the production of pro-inflammatory cytokines and promotes the production of anti-inflammatory cytokines. Scutellarin decreases the ROS and MDA concentrations, and increases the activity levels of antioxidative enzymes in serum and kidneys. Furthermore, its anti-inflammatory and antioxidative effects are mediated by the modulation of the Nrf2/HO-1 signaling pathway [29]. In the present study, we showed that scutellarin treatment resulted in significant decrease in Keap1 expression and increase in nuclear Nrf2 expression. Furthermore, scutellarin induced the expressions of two downstream genes of Nrf2/HO-1/ARE, HO-1, and NQO1, indicating that scutellarin regulates the Keap1/Nrf2/ARE signaling in H/R-induced hepatocytes. However, the precise regulatory mechanism underlying the induction effect of scutellarin on Keap1/Nrf2/ARE signaling needs further study.

In conclusion, our results demonstrated that scutellarin protected hepatocytes from H/R-induced cellular injury through regulating the Keap1/Nrf2/ARE signaling pathway. These findings indicated a potential relevance of scutellarin in attenuating hepatic H/R injury. However, further *in vivo* study is needed for the better understanding of the protective role of scutellarin in hepatic H/R injury.

## Supplementary Material

Supplementary Figures S1-S2

## Abbreviations

ARE: antioxidant-response element
DCFH-DA: 2′,7′-dichlorfluorescein diacetate
FITC: fluorescein isothiocyanate
4-HNE: 4-Hydroxynonenal
HO-1: heme oxygenase-1
H/R: hypoxia/reoxygenation
I/R: ischemia–reperfusion
Keap1: kelch like ECH associated protein 1
MDA: malondialdehyde
3-NT: 3-Nitrotyrosine
NAFLD: non-alcoholic fatty liver disease
NQO1: NAD(P)H:quinone oxidoreductase 1
Nrf2: nuclear factor erythroid 2-related factor 2
qRT-PCR: quantitative real-time PCR
ROS: reactive oxygen species
SOD: superoxide dismutase
