# Supplementary figures and images for: Scutellarin attenuates hypoxia/reoxygenation injury in hepatocytes by inhibiting apoptosis and oxidative stress through regulating Keap1/Nrf2/ARE signaling
Source: Biosci Rep. 2019 Nov 13;39(11):BSR20192501. doi: 10.1042/BSR20192501 (PMC6851522; doi:10.1042/BSR20192501)

**A**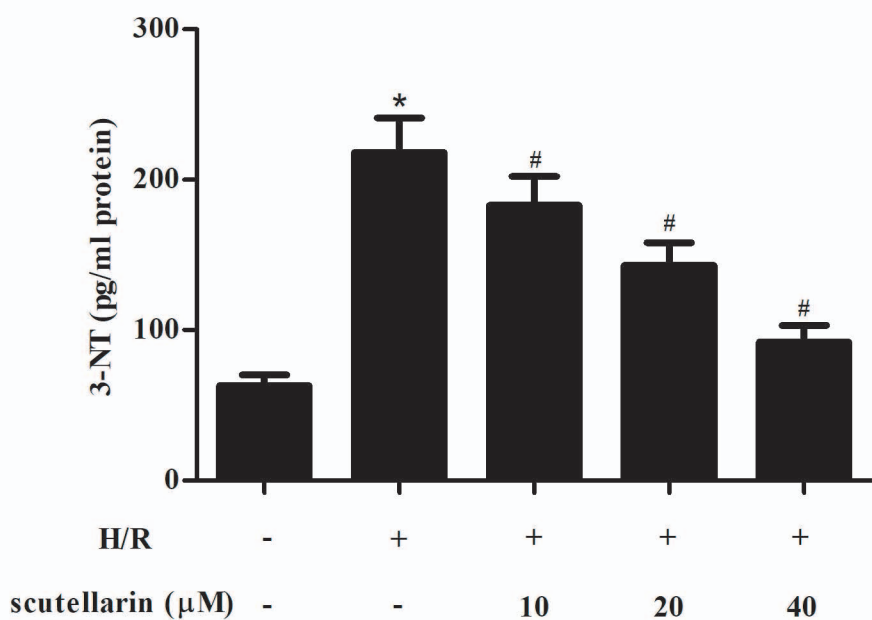**B**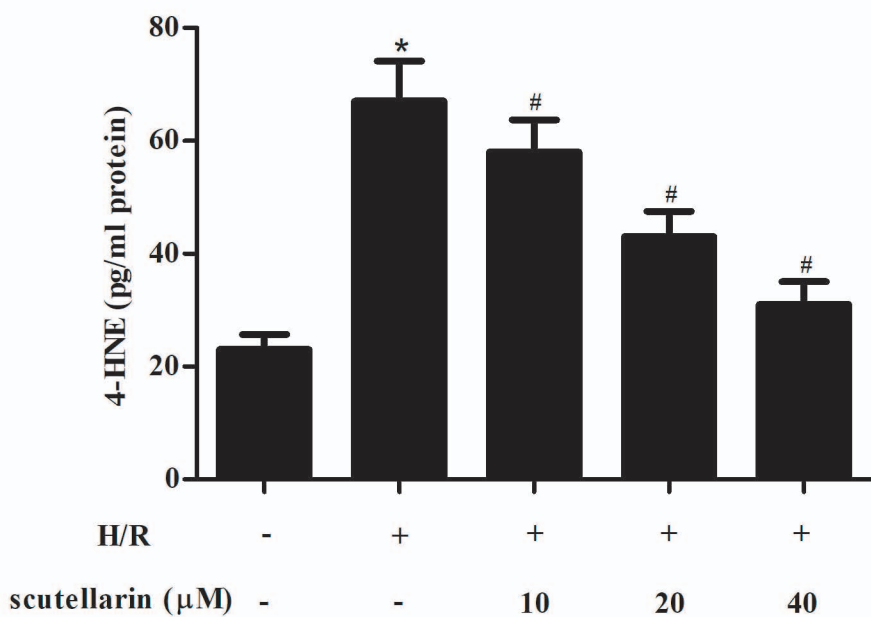

**A**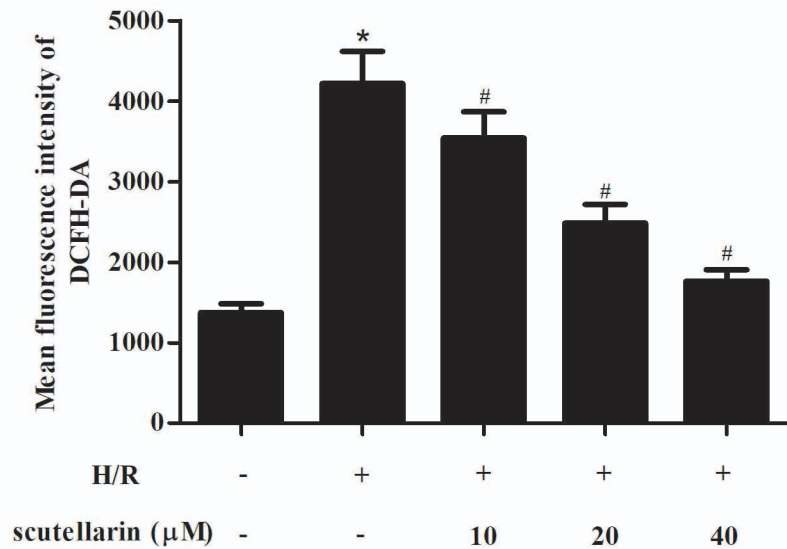**B**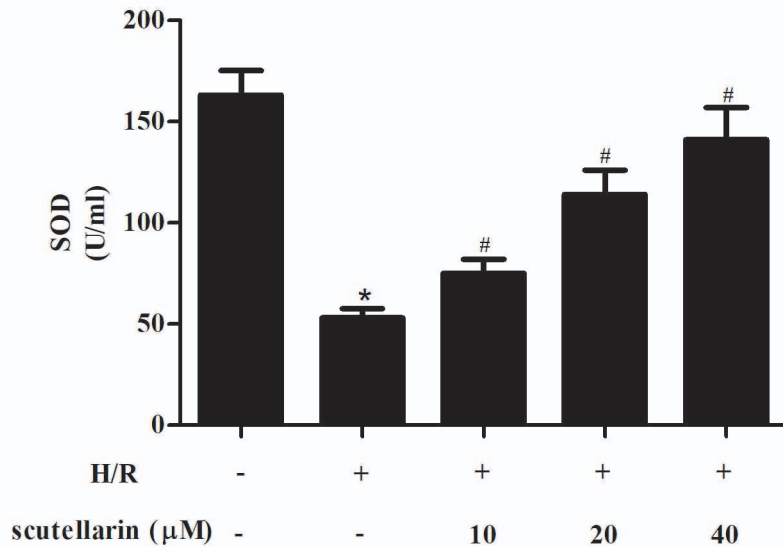

Supplement: Supplementary Figures S1-S2 [file BSR-2019-2501_supp.pdf]
